# A DROSOPHILA MODEL OF A MITOCHONDRIALLY INDUCED INNATE IMMUNE RESPONSE

**DOI:** 10.1093/geroni/igae098.2437

**Published:** 2024-12-31

**Authors:** Adrienne Wang, Angshita Dutta, Alexandra Putzier, Marieke Trabant, Kyla Gustave-Schelling, Monica Sanchez-Contreras, Scott kennedy

**Affiliations:** Western Washington University, Bellingham, Washington, United States; Western Washington University, Bellingham, Washington, United States; Western Washington University, Bellingham, Washington, United States; Western Washington University, Bellingham, Washington, United States; Western Washington University, Bellingham, Washington, United States; University of Washington, Seattle, Washington, United States; University of Washington, Seattle, Washington, United States

## Abstract

Mitochondrial function and innate immunity-mediated inflammation are two critical regulators of healthy aging and lifespan. While mitochondrial function is the cornerstone of some of the oldest theories of aging, innate immune function is increasingly implicated as a modifier of age, and age-related neurodegenerative diseases. There is mounting evidence that mitochondria play a role in regulating innate immune function and inflammation, and that mitochondrial dysfunction and mitochondrial DNA (mtDNA) stress can lead to increased innate immune signaling. While severe mtDNA depletion is lethal, studies have shown that moderate depletion (50%) of mtDNA in mice induces a mitochondrial stress response that upregulates innate immune signaling. This signaling depends on release of mtDNA into the cytoplasm, and results in an increase in viral resistance however, the exact mechanisms by which mitochondrial stress leads to innate immune activation and subsequent effects on lifespan and susceptibility to neurodegenerative disease are not well understood. Here, we present a Drosophila model of mtDNA depletion in which expression of UL12.5, a nuclease expressed by the Herpes simplex-1 virus, degrades mtDNA. Expression of UL12.5 is developmentally lethal in an array of tissues, and conditional depletion of mtDNA in adult flies leads to decreased lifespan, exacerbates toxicity of the AD-related protein Tau, and induces an innate immune response that leads to increased resistance to acute bacterial challenge. This model provides an opportunity to extend our understanding of the role of sterile inflammation and mitochondrial stress in the etiology of age-related changes in immune function and susceptibility to neurodegenerative disease.

